# A Case of Inadvertent Overdose of Vosoritide Injection in a Three-Month-Old Infant With Achondroplasia

**DOI:** 10.7759/cureus.114690

**Published:** 2026-08-17

**Authors:** Shunya Satake, Yoichiro Oda

**Affiliations:** 1 Department of Pediatrics, Chigasaki Municipal Hospital, Chigasaki, JPN

**Keywords:** achondroplasia, drug overdose, infant, safety, vosoritide

## Abstract

Vosoritide, a recombinant C-type natriuretic peptide analog that antagonizes fibroblast growth factor receptor 3 (*FGFR3*) signaling, is recommended for early initiation in infants with achondroplasia. However, clinical safety data for infants under six months remain limited, and concerns persist regarding its potential to induce symptomatic hypotension. We present a case of a three-month-old female infant with genetically confirmed achondroplasia (*FGFR3* p.Gly380Arg) who accidentally received an inadvertent double dose of vosoritide (0.24 mg; 53 μg/kg) during home self-injection therapy. This patient remained completely asymptomatic, exhibiting no signs of hypotension, lethargy, or facial pallor during home monitoring. While findings from a single patient cannot definitively establish safety, the complete absence of adverse reactions after an accidental overdose suggests substantial inter-individual variability in hemodynamic sensitivity to vosoritide during early infancy. This clinical observation provides insights into the drug’s tolerability profile in young infants.

## Introduction

Achondroplasia is the most common form of disproportionate short stature caused by a constitutive gain-of-function mutation in the fibroblast growth factor receptor 3 (*FGFR3*) gene [1]. This variant overactivates downstream signaling pathways that inhibit chondrocyte proliferation and differentiation in the growth plate, thereby impairing endochondral ossification. Vosoritide, a modified recombinant C-type natriuretic peptide (CNP) analog, was developed as a first-in-class targeted therapy that antagonizes *FGFR3* signaling. A primary safety concern of vosoritide is its pharmacological effects on the cardiovascular system [2]. As a CNP analog, vosoritide induces vasodilation through natriuretic peptide receptor-B activation, potentially leading to symptomatic hypotension.

The 2025 international consensus guidelines on the implementation and monitoring of vosoritide therapy in individuals with achondroplasia now recommend the initiation of vosoritide as early as possible to maximize clinical benefits, such as improved skeletal proportionality and potential reduction in complications such as foramen magnum stenosis [3]. The recommended dose of vosoritide is 15 μg/kg for patients aged two years or older and 30 μg/kg for those under two years of age. However, clinical evidence regarding the efficacy and safety of vosoritide in infants aged <6 months remains limited.

We previously reported two cases of infants who experienced cardiovascular adverse events following standard doses of vosoritide [4]. In this report, we describe a rare case of a three-month-old infant who accidentally received an almost double dose (53 μg/kg) of vosoritide and remained entirely asymptomatic.

## Case presentation

**Figure 1:**
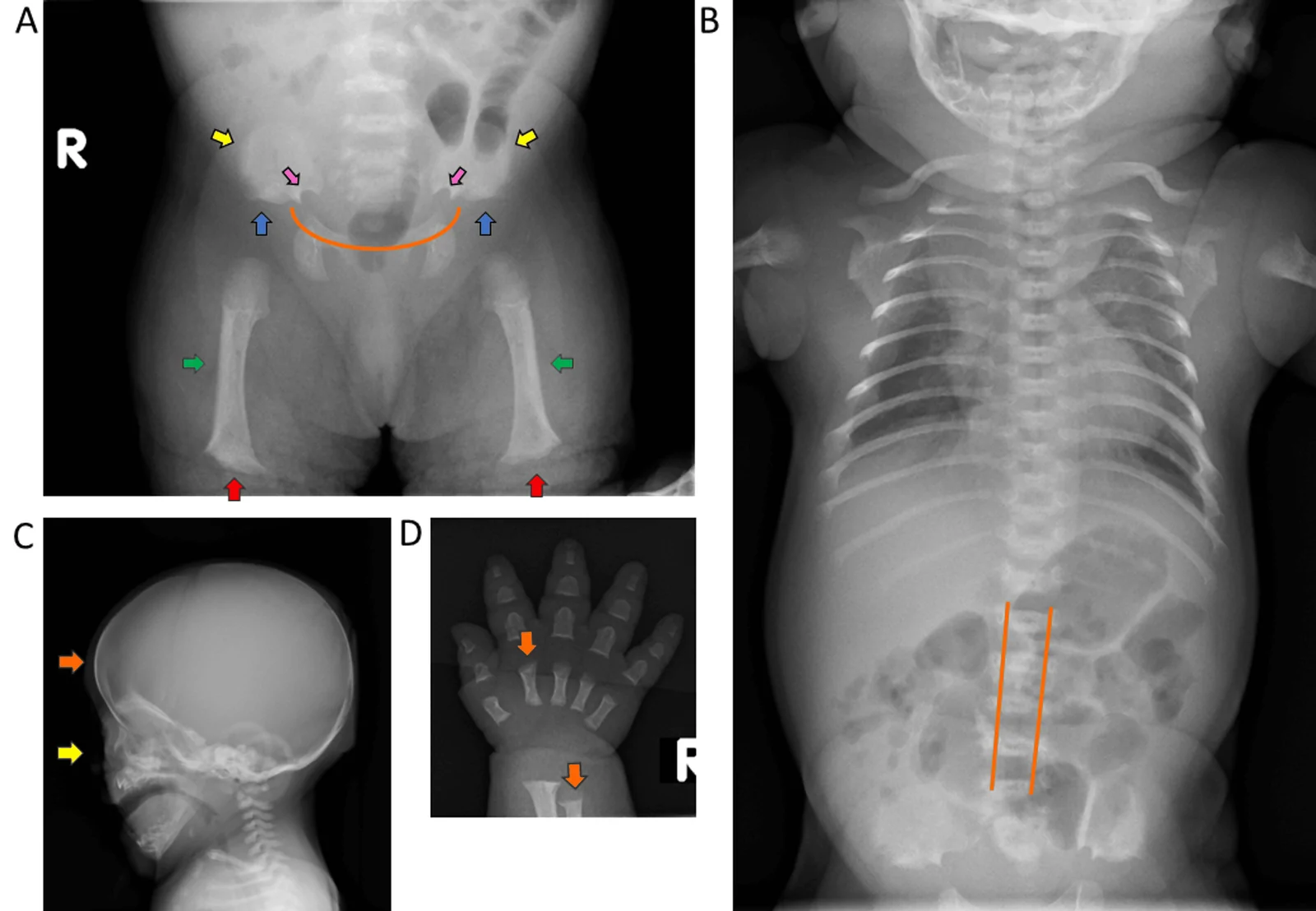
Radiographic findings in the neonatal period. (A) Pelvis and femurs: Anteroposterior view demonstrating a “champagne glass” pelvic inlet (orange line), squared iliac wings (yellow arrows), horizontal acetabular roofs (blue arrows), and narrow sacrosciatic notches (pink arrows). Pronounced rhizomelic shortening of the femurs (green arrows) is observed, accompanied by metaphyseal flaring and cupping (red arrows). (B) Spine: Anteroposterior view showing the interpedicular distance slightly narrowing from L1 to L5 (orange lines). (C) Skull: Findings include prominent frontal bossing (orange arrow) and midface hypoplasia (yellow arrow). (D) Right hand: Characteristic metaphyseal flaring and cupping of the tubular bones (orange arrows).

A one-month-old Japanese female infant with achondroplasia was referred to our hospital for the initiation of vosoritide therapy. Her clinical history was notable for prenatally identified shortened limbs on fetal ultrasonography. She was delivered via elective cesarean section because of a breech presentation. Birth growth parameters were as follows: weight, 3,292 g (+1.6 standard deviation (SD)); length, 42 cm (−2.9 SD); and head circumference, 38 cm (+3.83 SD). Postnatal skeletal radiography revealed features consistent with achondroplasia (Figure 1). Diagnosis was confirmed using genetic testing, which identified a heterozygous pathogenic variant of the *FGFR3* gene (p.Gly380Arg).

At initial presentation to our hospital, the patient’s growth parameters were as follows: length, 45.8 cm (−3.2 SD); and weight, 3.585 g (−1.0 SD). Physical examination revealed marked shortening of the extremities, particularly in the proximal segments (rhizomelic shortening), and redundant skin folds. Additionally, she exhibited craniofacial features characteristic of achondroplasia, including macrocephaly, frontal bossing, and midface hypoplasia.

At two months of age, daily home self-injection of vosoritide was initiated. The medication was administered at a dose of 0.12 mg (30 μg/kg) in strict accordance with the weight-based dosage in the prescription information. The family was instructed to give the injection one hour after feeding to mitigate hypotensive risks [4]. A single episode of post-injection emesis occurred. No symptoms suggestive of hypotension were reported.

At three months of age, the family inadvertently administered a full vial (i.e. 0.24 mg (53 μg/kg) instead of 0.12 mg. Upon receiving a notification by telephone, the medical team assessed the risk. Since the patient was 30 minutes away from an emergency facility and had already passed the 10-minute mark without symptoms, the family was instructed to closely monitor the patient for one hour--a duration determined based on the drug's rapid elimination profile (time to maximum concentration, 14.4 minutes; elimination half-life, 27.0 minutes) [5]. During this period, particular attention was directed toward signs suggestive of hypotension, such as vomiting, facial pallor, or lethargy. According to the family, the patient remained completely asymptomatic, showing no observable signs of hypotension or skin reaction at the injection site. Ultimately, the family chose not to visit the emergency department.

Following this episode, the vosoritide therapy was continued, during which several additional episodes of emesis occurred without any further symptoms associated with hypotension.

## Discussion

We previously reported two cases of infants with cardiovascular adverse events following subcutaneous administration of vosoritide at one and three months of age [4]. In contrast, the current patient exhibited no adverse reactions even with a double-dose injection at three months of age, suggesting substantial inter-individual variability in pharmacological sensitivity. This is consistent with the findings of Chan et al., who reported no significant correlation between maximum plasma concentration and the magnitude of blood pressure reduction [5]. Our clinical observations further corroborate this evidence.

According to an initial report from an international phase 2 clinical study (Study 111-202) involving patients with achondroplasia aged 5-14 years, vosoritide was administered subcutaneously once daily at doses of 2.5, 7.5, 15.0, and 30.0 μg/kg. Results indicated that their pharmacodynamic response, as measured by cyclic guanosine monophosphate activity (a marker of CNP activity), reached saturation at 15.0 μg/kg; consequently, this dosage was maintained for subsequent evaluations [2].

Furthermore, a phase 2, double-blind, randomized, placebo-controlled trial was conducted in children with achondroplasia aged 3-59 months. In this study, infants aged 0-23 months received 30.0 μg/kg of vosoritide, whereas children aged 24-59 months were administered a dose of 15.0 μg/kg [6]. The higher dosage requirement for vosoritide in patients aged <2 years is attributable to the increased weight-normalized clearance observed in this age group [5].

The 2025 international consensus guidelines do not require home blood pressure monitoring but emphasize pre-dose hydration and educating families on recognizing signs of hypotension such as cold extremities or somnolence [3]. Furthermore, it recommends the initiation of vosoritide as early as possible to maximize clinical benefits, such as improved skeletal proportionality and potential reduction in complications such as foramen magnum stenosis. However, in the international trial involving children aged 3-59 months, the mean age at treatment initiation was 5.66 ± 0.44 months (youngest: 4.4 months of age), with a sample size of only nine patients [6]. Consequently, clinical evidence regarding the efficacy and safety of vosoritide in infants aged <6 months remains limited.

Moreover, this case highlights the importance of caregiver education regarding accurate dose preparation, recognition of potential adverse effects, and prompt communication with the treating medical team after any dosing error.

Although our findings are based on a single patient and asymptomatic tolerance does not imply overdose safety, this clinical observation may contribute to a better understanding of the safety profile of this population. Given that vosoritide has been approved across all age groups in Japan, Australia, and the United States, results from post-marketing surveillance are expected to substantiate the safety and tolerability of vosoritide in early infancy.

## Conclusions

The absence of adverse reactions in our patient, even after the administration of an almost double dose at three months of age, suggests substantial inter-individual variability in hemodynamic sensitivity to vosoritide during early infancy. Furthermore, this case highlights the importance of caregiver education regarding accurate dose preparation, recognition of potential adverse effects, and prompt communication with the treating medical team after any dosing error. With the increasing global adoption of vosoritide in infants aged <6 months with achondroplasia, further clinical data are essential to ensure pharmacological safety.
